# Metabolic Specialization and Synergy in Alkane Degradation By a Microbial Consortium: An Integrated Genomic and Geochemical Approach

**DOI:** 10.1007/s00284-026-04858-6

**Published:** 2026-03-27

**Authors:** Pedro Guilherme Barreto de Santana Nazaré, Adonilson Alves de Menezes Neto, Letícia Araújo de Oliveira, Antônio Fernando de Souza Queiroz, Olívia Maria Cordeiro de Oliveira, Danusia Ferreira Lima

**Affiliations:** Instituto de Geociências, UFBA - Av. Milton Santos, s/n° - Sala 303-A - Ondina, Salvador, 40170-110 BA Brasil

## Abstract

The constant demand for petroleum and the recurrence of environmental accidents led to the development of sustainable strategies for the treatment of contaminated areas, such as bioremediation. Alkanes, being one of the most abundant and challenging fractions of petroleum in terms of biodegradation, demand microorganisms with high metabolic specialization and the incorporation of bacterial consortia, which possess high efficiency due to inter-species synergy. This study had as its objective to integrate genomic and geochemical analyses to unveil the potential and mechanisms of alkane degradation by nine bacterial strains (*Bacillus*, *Brevibacillus*, *Pseudomonas*, and *Stenotrophomonas*) isolated from a contaminated mangrove. The genomic sequencing and functional annotation revealed the presence of key genes and enzymes associated with alkane activation and oxidation, distributed among different bacterial genera. The geochemical analyses by GC-FID, supported by statistical evaluation, demonstrated the obligatory synergy of the complete consortium for the efficient degradation of intermediate fractions. Individual strains exhibited distinct ecological roles: *Pseudomonas* and *Stenotrophomonas* acted as rapid degraders, while *Bacillus* specialized in the delayed and highly efficient degradation of recalcitrant heavy fractions, likely mediated by biosurfactant production. Furthermore, the study evidenced the complexity of geochemical data analysis in multiphasic systems, where anomalous negative variations were identified as methodological artifacts resulting from biofilm formation and heterogeneous sampling, rather than metabolic limitations. The integration of genomic and geochemical results revealed itself to be a powerful tool to elucidate microbial interactions and to understand the specific alkane degradation mechanisms of each genus in the bioremediation process.

**Graphical Abstract:**

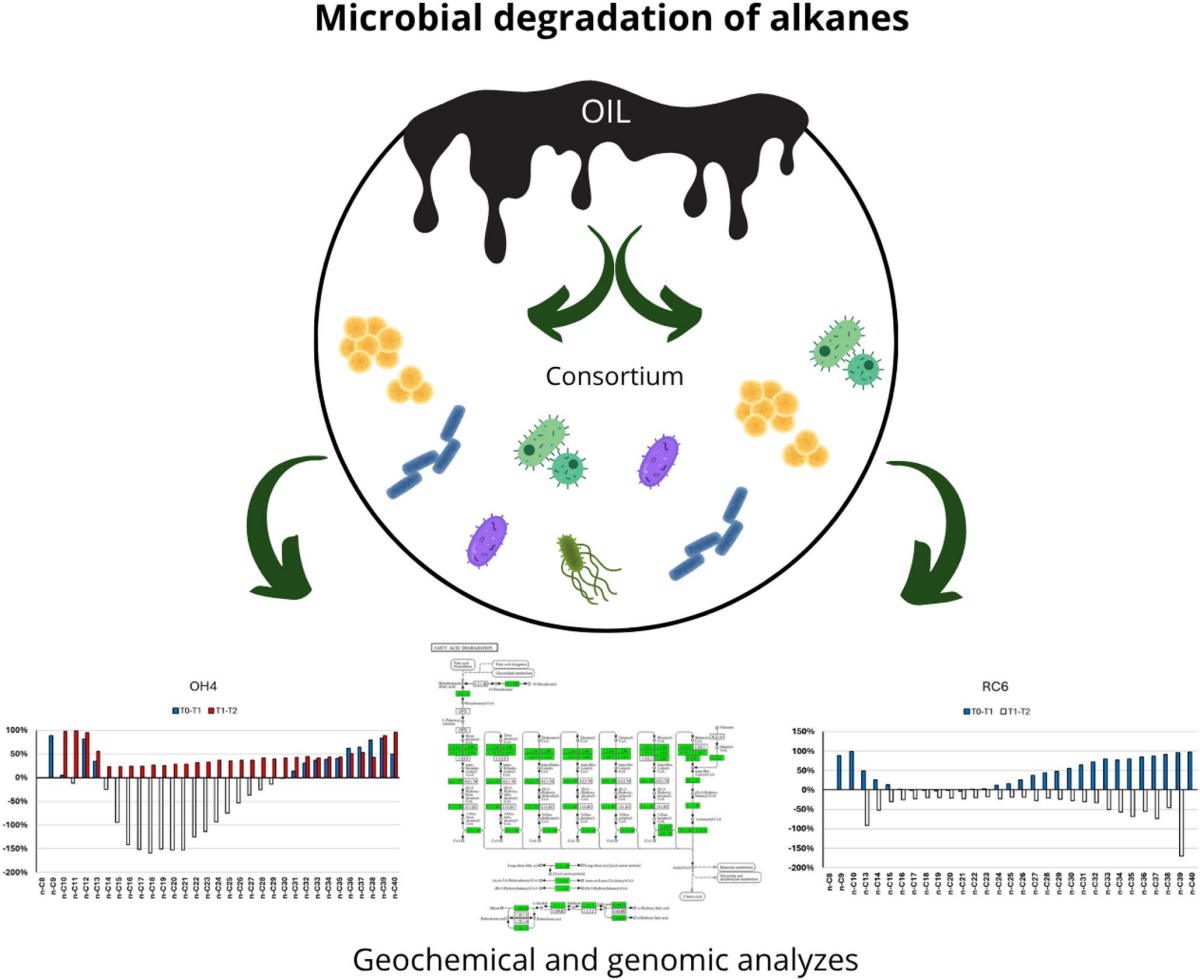

## Introduction

Bioremediation has established itself, since the 1980 s, as a sustainable and effective alternative for the treatment of environments contaminated by organic and inorganic pollutants. This approach gained notoriety after its application in the Exxon Valdez oil tanker disaster, which occurred in 1989, that evidenced the potential of microorganisms in the degradation of complex and persistent contaminants, such as crude oil [3, 42].

Since then, the increase in global demand for petroleum and its derivatives, estimated at up to 110 million barrels per day by 2045, according to OPEC, and the growing occurrence of spills during prospecting, transport, and refining have reinforced the need for ecological and economically viable strategies to mitigate the environmental impacts of these accidents [33].

Thus, bioremediation which utilizes the metabolic capacity of autochthonous microorganisms to degrade, detoxify, or transform petroleum compounds, represents an efficient, sustainable, and low-cost solution, especially when compared with traditional physical and chemical methods [14, 37, 38]. This process is based on complex enzymatic cascades, in which hydrocarbons are converted into less toxic intermediates and, finally, used as a source of carbon and energy [2].

Among the main constituents of petroleum, alkanes stand out for being a major fraction and for the structural variability that directly influences their biodegradability. Although short and medium-chain alkanes are generally more susceptible to microbial oxidation, long-chain ones present greater recalcitrance due to low solubility and enzymatic accessibility [27]. The degradation of these compounds involves specific metabolic routes initiated by the action of monooxygenases and dehydrogenases, culminating in the conversion of alkanes into alcohols, aldehydes, and, subsequently, into fatty acids that integrate the β-oxidation cycle [8, 40].

The diversity of these routes is potentiated in microbial consortia, symbiotic systems formed by different bacterial species with complementary metabolic capacities. This cooperation increases degradation efficiency, broadens tolerance to pollutant toxicity, and favors the production of compounds such as biosurfactants, which reduce surface tension and facilitate access to hydrophobic hydrocarbons (Parus et al., 2023) [23].

Despite advances, important gaps still persist in the understanding of the molecular mechanisms that sustain alkane biodegradation, especially regarding the interaction between species in a consortium and the regulation of the enzymatic routes involved [5, 30]. In this sense, omics technologies, such as functional genomics, have shown themselves to be fundamental tools for the elucidation of metabolic pathways and the specific contribution of each microorganism within complex communities [1].

In this way, the present study aimed to integrate genomic and geochemical analyses to investigate the alkane degrading potential in bacterial strains isolated from a mixed consortium originating from a petroleum-contaminated mangrove. Through the combination of functional analyses and experimental evaluation, it was sought to understand the metabolic routes involved in alkane degradation and to identify the main microorganisms and enzymes associated with this process, contributing to the development of more efficient strategies in the bioremediation of aliphatic hydrocarbons.

##  Materials and Methods

### Biological Material

The microorganisms used in this study are part of a microbial consortium composed of 36 organisms, isolated in the Baía de Todos os Santos and protected by the patent of Lima et al., [19], with deposit number at the National Institute of Industrial Property (INPI) BR 10 2021 002341 4. For the present investigation, 9 bacterial strains were selected based on taxonomic diversity and preliminary metabolic profile. The taxonomic identifications were performed from the analysis of the 16 S rRNA region, associated with classic microbiology techniques, and the respective genera are listed in Table 1.

**Table 1:**
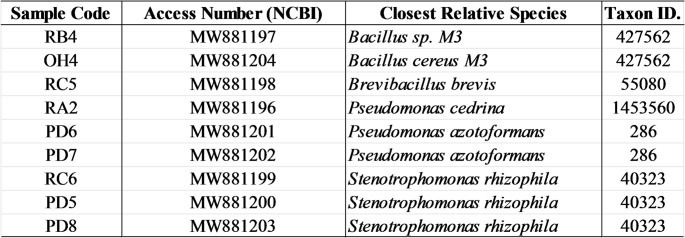
Taxonomic identification of the bacterial strains selected for genomic analysis

#### Experimental Configuration

For this study, an assay was designed to analyze the degradation of hydrocarbons by the bacterial isolates with 3 distinct growth conditions:

- Standard condition: microorganisms cultivated in standard nutritive medium, destined for genomic analyses;
- Experimental condition: cultivation with crude oil as the only carbon source, simulating a contaminated environment;
- Negative control (blank): medium containing only the crude oil, without bacterial inoculation, to evaluate the abiotic degradation of the petroleum.

### Genomic Sequencing and Data Processing

#### Genome Sequencing

The genomic sequencing of the strains was performed by a third-party service at the University of Göttingen, Germany, using the Illumina Hi-Seq 2500 platform, with generation of 2 × 150 bp paired-end reads (500 bp insert). The quality control of the raw reads was performed with the FastQC software adopting a phred in the 30 s range, followed by low-quality filtering, adapter removal and end trimming by means of the fastp tool [32]. The genome assembly was performed with Unicycler, and the evaluation of assembly metrics, such as N50, number of contigs and coverage, was conducted with QUAST [10].

#### Genomic Annotation

The annotation of the bacterial genomes was performed in a combined manner using the RASTtk (v2.0) pipeline, accessed via the BV-BRC (v3.31.15) Comprehensive Genome Analysis service, and Prokka (v1.14.6). For Prokka, protein-coding genes (CDS) were predicted using Prodigal (v2.6.3), while tRNAs and rRNAs were identified using Aragorn (v1.2.41) and Barrnap (v0.9), respectively. Functional annotation was conducted through a hierarchical search against IS, AMR, UniProtKB/Swiss-Prot, and HAMAP databases using BLAST+ (v2.16.0) and HMMER (v3.4), with an e-value cutoff of 1e-09 and minimum coverage of 80%. Non-coding RNAs were identified using Infernal (v1.1.5). The RASTtk functional analysis was based on the SEED subsystems. The integrated annotation was submitted to GhostKOALA for KO number attribution, followed by pathway reconstruction using KEGG Mapper, simultaneously with comparison and validation via the PATRIC platform. Furthermore, the annotations were enriched with specialized databases, including UniProt, InterProScan, Gene Ontology (GO), and BRENDA, to maximize accuracy in identifying gene functions.

#### Comparative Analysis

From the annotated data, a functional analysis was performed to identify genes and metabolic pathways associated with the biodegradation of aliphatic hydrocarbons, specifically targeting the fatty acid degradation pathway (KEGG map00071). Comparative analysis focused on key enzymes such as monooxygenases (alkB, ladA, almA), cytochromes P450, and β-oxidation enzymes. Promoter region predictions were performed with BPROM (Softberry). To resolve taxonomic ambiguities and refine isolate identification, a phylogenomic analysis was conducted. A codon-based phylogenetic tree was constructed using the BV-BRC platform; closely related genomes were identified using MASH, and the final phylogeny was built with RAxML (v8.2.11) using 100 rapid bootstrap replicates. The substitution models utilized were JTT for amino acid sequences and GTR for nucleotide sequences. Data were integrated via BV-BRC visualization tools and organized into heatmaps and comparative tables to interpret the functional organization and biodegrading potential of the consortium.

### Geochemical Experiment

#### Experimental Setup

The microorganisms used in the experiments were cultivated in Petri dishes for 48 h. Then, they were transferred to 250 mL Erlenmeyer-type flasks containing liquid culture medium, with the objective of cultivating the isolates. After growth, the isolates were transferred to 25 mL of saline solution, and their concentrations were standardized using a microplate reader (Loccus). The microgeological experiment was set up in four 500 mL Erlenmeyer-type flasks containing: 300 mL of Bushnell-Haas Broth (BH) culture medium, poor in carbon; 9 mL of the standardized saline solution containing the bacterial strain in the experimental case; and 3 g of the carbon source.

The crude oil used was supplied by the independent operator of onshore oil and gas exploration and production Petrorecôncavo S.A., Field: Norte Fazenda Caruaçu – Recôncavo Basin, Depth: 1064.0 m, Density @ 20 °C: 0.8371, °API @ 20 °C: 37.36. It is a stabilized paraffinic oil, stored long-term under anhydrous conditions from the Recôncavo Basin, Bahia - Brazil, The Erlenmeyer-type flasks were then incubated at 30 °C for a period of 31 days, during which oil samples were collected at times 0 h (T0), 16 days (T1), and 31 days (T2). The samples were dissolved in dichloromethane (DCM), filtered through an open column of activated sodium sulfate, and stored.

#### Analytical Methods

The Total Petroleum Hydrocarbons were analyzed by GC/FID on an Agilent 7890B chromatograph, split method, whose usage specifications are described in Table 2. The quantification of the compounds was performed by the external standard method, based on peak area, within the time interval of 2.5 to 40 min. The data were acquired and processed using the ChemStation software (Agilent Technologies), with the sensitivity and rejection parameters adjusted to guarantee the accuracy in the identification and quantification of peaks.

**Table 2:**
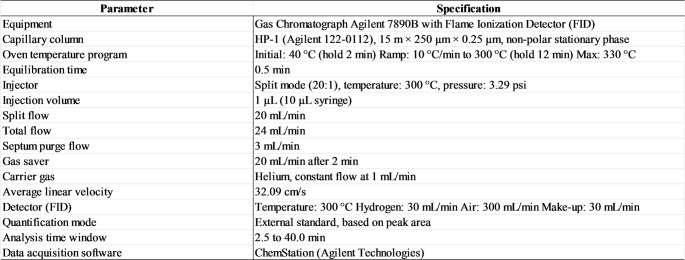
Parameters of the chromatographic analysis by GC/FID

Finally, a statistical analysis of the chromatographic data was performed using the degradation rate (%) as the response variable. To evaluate metabolic specialization and provide statistical robustness, the quantified n-alkanes (from n-C8 to n-C40) were grouped into three distinct structural fractions: Light (C8–C14), Intermediate (C15–C28), and Heavy (C29–C40). Because the experimental design utilized single batch bioreactors for each treatment, the individual aliphatic compounds within each defined fraction were treated as statistical replicates. This approach captures the variance associated with the metabolic selectivity of the strains across different carbon chain lengths, rather than experimental error.

To ensure the reliability of the trends, anomalous negative values identified as sampling artifacts were mathematically treated as zero (negligible degradation) prior to rate calculations, minimizing the impact of physical heterogeneity on the quantitative analysis. Degradation rates were evaluated both cumulatively (0–31 days) and dynamically for the late experimental phase (16–31 days). Normality was verified by means of the Shapiro-Wilk test, followed by an analysis of variance (ANOVA) to compare the performance between microbial treatments. When statistically significant differences were observed, Tukey’s HSD test was applied as a post-hoc for the identification of multiple comparisons between groups. All tests were conducted with a significance level of α = 0.05, using the R statistical software environment.

## Results and Discussion

### Complete Genome Sequencing and Comparative Analysis

Complete genomic sequencing was performed for the nine bacterial strains integrating the microbial consortium, generating high-quality data and adequate coverage for de novo assembly of the genomes (Table 3). The quality control and assembly steps were conducted using the FastQC, Fastp, and Unicycler tools, and evaluated by means of QUAST, as described in the methodology.

**Table 3:**
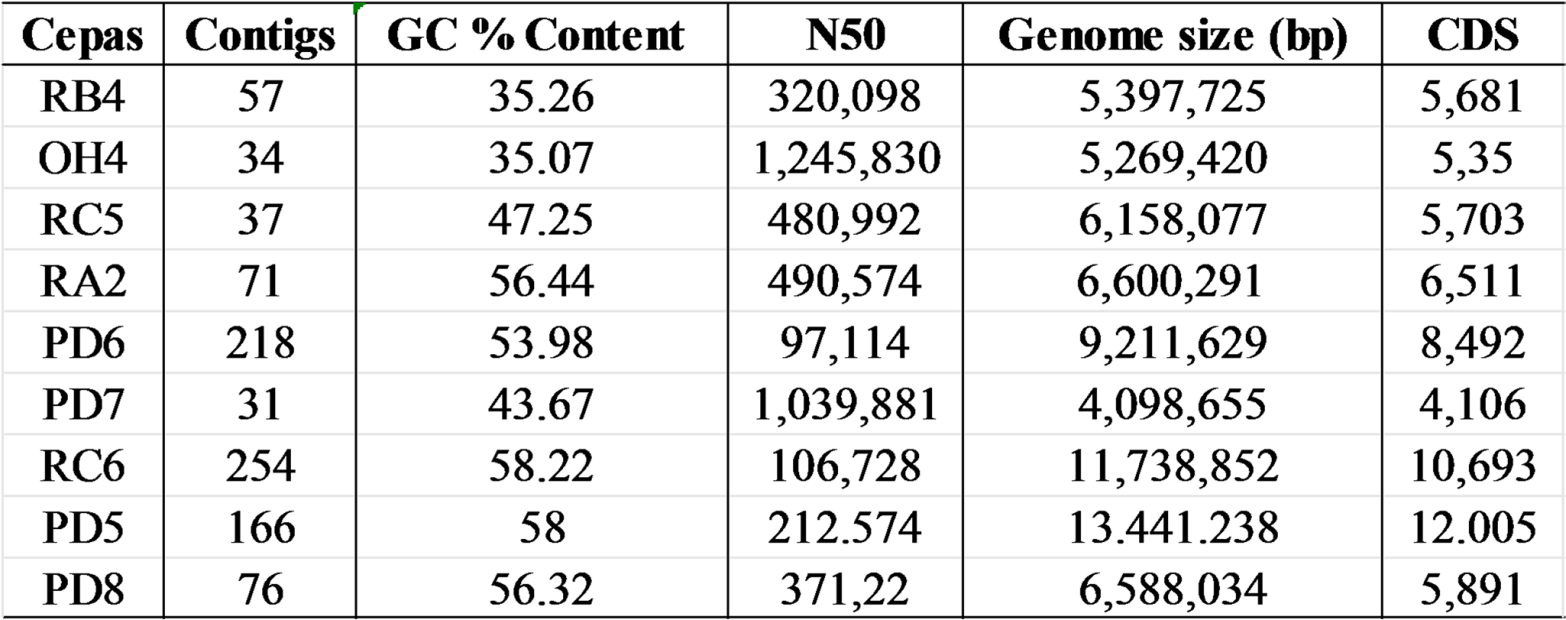
Quality parameters of the sequencing and assembly of the bacterial genomes

The assembled genomes presented sizes varying from 4.09 to 13.44 Mbp, with GC content between 38.2% and 67.5%, and total number of CDS between 3,950 and 12,400, values compatible with those expected for environmental bacteria with broad metabolic plasticity. The variation observed between the genera reflects the structural diversity of the consortium, suggesting the coexistence of microorganisms with different physiological and metabolic strategies.

The number of contigs varied from 31 to 254, with the lowest values observed in strains of the *Bacillus* and *Brevibacillus* genera, which indicates greater continuity and assembly quality—reinforced by the N50 values. This structural heterogeneity is consistent with data from environmental bacterial genomes [28, 36], and may be associated with the presence of mobile genetic elements and repetitive regions that hinder genomic closure in short-read sequencing [25].

In general, the obtained assemblies were sufficiently robust for functional analyses and metabolic pathway reconstruction, allowing the identification of key genes associated with hydrocarbon degradation. The observed genomic diversity, reflected in the GC content and genetic size, reinforces the cooperative potential of the consortium in contaminated environments, since different physiological profiles favor adaptation to variable conditions and the functional division of catabolic routes [26, 31] .

#### Alkane Metabolization Pathway

**Fig. 1:**
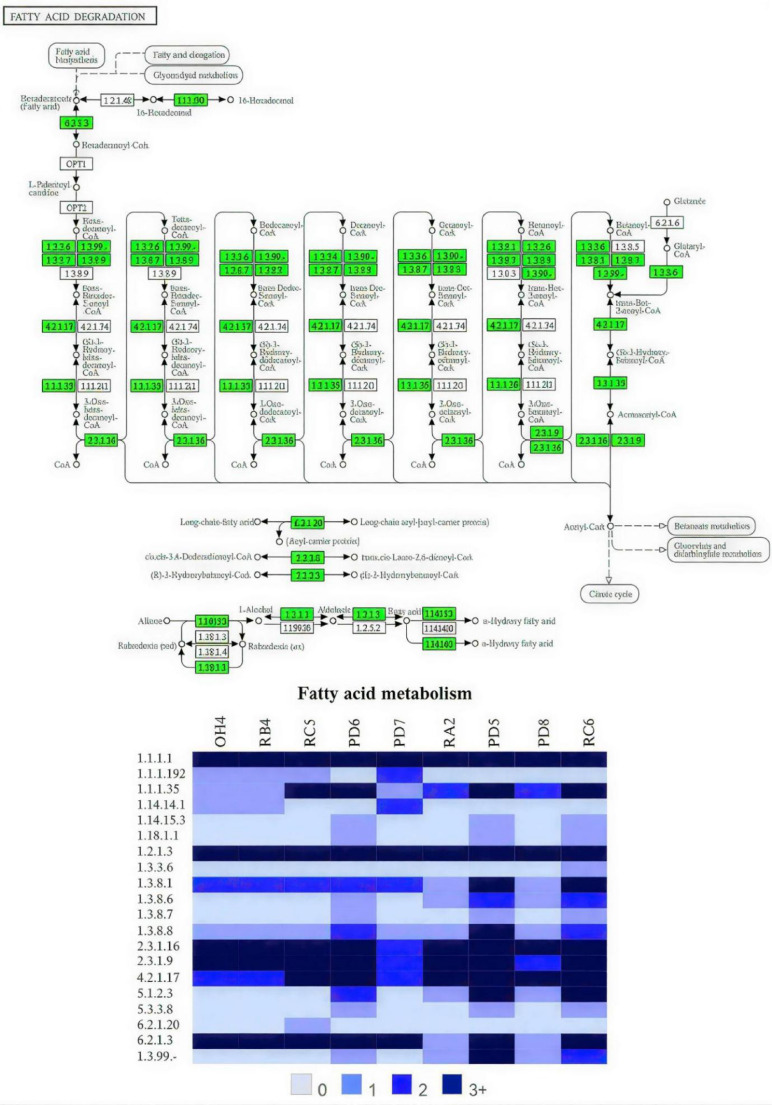
KEGG map00071 (fatty acid metabolism) for the microbial consortium

The functional reconstruction of metabolic pathways revealed the broad representation of the fatty acid β-oxidation route (KEGG map00071) among the nine bacterial strains of the consortium. The mapping of EC numbers (Fig. 1) indicated the complete presence of the main enzymes involved in the oxidative catabolism of alkanes, including acyl-CoA synthetase (EC 6.2.1.3), acyl-CoA dehydrogenase (EC 1.3.8.1), enoyl-CoA hydratase (EC 4.2.1.17), 3-hydroxyacyl-CoA dehydrogenase (EC 1.1.1.35), and thiolase (EC 2.3.1.16). These enzymes participate in the sequential steps of oxidation and cleavage of fatty acids down to acetyl-CoA, evidencing the complete functioning of the downstream β-oxidation route.

Furthermore, genes related to the initial activation of alkanes were identified, such as alkane 1-monooxygenase (EC 1.14.15.3), Cytochrome P450 (1.14.14.1), alcohol dehydrogenase (EC 1.1.1.1), and aldehyde dehydrogenase (EC 1.2.1.3), responsible for the conversion of alkanes into alcohols and subsequently into fatty acids. These results indicate a complete functional arrangement, spanning from the initial oxidation to the mineralization of the aliphatic compounds [9, 41].

The consortium demonstrated the presence of genes for different monooxygenases, including those specific for short chains, such as AlkB (alkB1_2, alkM), and the more versatile ones, such as Cytochrome P450 (102A3, 102A2, 102A5), both responsible for the terminal hydroxylation of linear alkanes. In addition to those of β-oxidation distributed among the different strains, suggesting interspecies metabolic cooperation and the existence of a biochemical strategy for the catabolism of aliphatic compounds present in petroleum. The lineages of the *Pseudomonas* and *Stenotrophomonas* genera (PD5, PD6, RC6) were highlighted, due to the presence of alkB, constantly used as a parameter for evaluating the alkane degradation potential in biodegrading organisms, while Cytochrome CYP102A5 is normally associated with the *Bacillus* genus (OH4 and RB4), as a versatile and promiscuous enzyme in its targets, capable of metabolizing non-natural substrates, including polyethylene film [11, 21, 34, 39].

The redundancy of genes associated with alkane metabolism in multiple strains, combined with the presence of promoters and regulatory elements linked to oxidative stress response, suggests an evolutionary adaptation to the contaminated environment, in which continuous exposure to hydrocarbons favored the selection of specialized lineages [4]. This scenario reinforces the hypothesis of functional distribution of catabolic routes within the consortium, maximizing its degrading efficiency.

### Total Petroleum Hydrocarbons

**Fig. 2:**
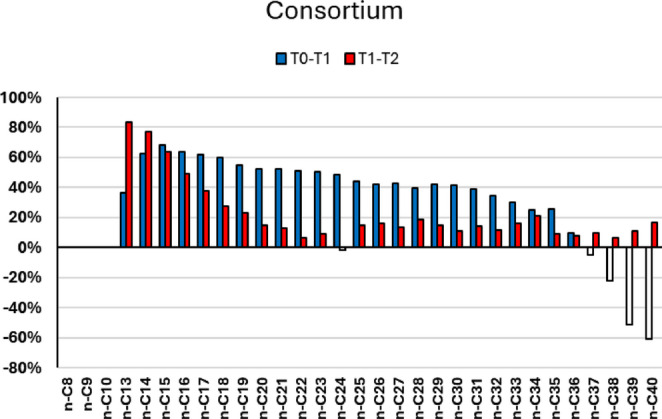
Degradation rate of total petroleum hydrocarbons in the medium inoculated with complete consortium under standardized conditions

For the comparative analysis of the individual degradative potential of the studied strains, previous results representative of the degradative capacity of the complete consortium under standardized conditions were used. In conjunction with the negative control, in which there was no inoculation, the use of a stabilized paraffinic oil stored long-term under anhydrous conditions to limit the presence of active indigenous microbial populations, made it possible to infer that any contrast obtained between the samples resulted directly from microbial action. Furthermore, as all experimental conditions were standardized regarding the initial petroleum and inoculum concentrations, a direct comparison between the treatments becomes viable (Fig. 2).

The analysis of the microbial consortium revealed a high degradation rate throughout the 31-day experiment, standing out for its capacity to act rapidly across all structural fractions of the crude oil. The consortium reached a general average degradation of 47.76%, achieving 52.24% specifically in the intermediate fraction within the first 16 days. This early efficiency highlights the fundamental role of consortia in the synergistic degradation of hydrocarbons when nutrient availability is high and toxic metabolite accumulation is low [43]. However, in the last 15 days, the degradation rate for the intermediate fraction decreased to 29%. This reduction likely reflects the accumulation of inhibitory metabolic intermediates, such as alcohols and aldehydes, characteristic of closed batch systems [6]. Considering the sequential nature of biodegradation, progressing from highly bioavailable light chains to recalcitrant heavy ones, an extended timeframe coupled with nutrient replenishment would likely prevent this inhibition, sustaining the continuous degradation of the heavier fractions [44].

**Fig. 3:**
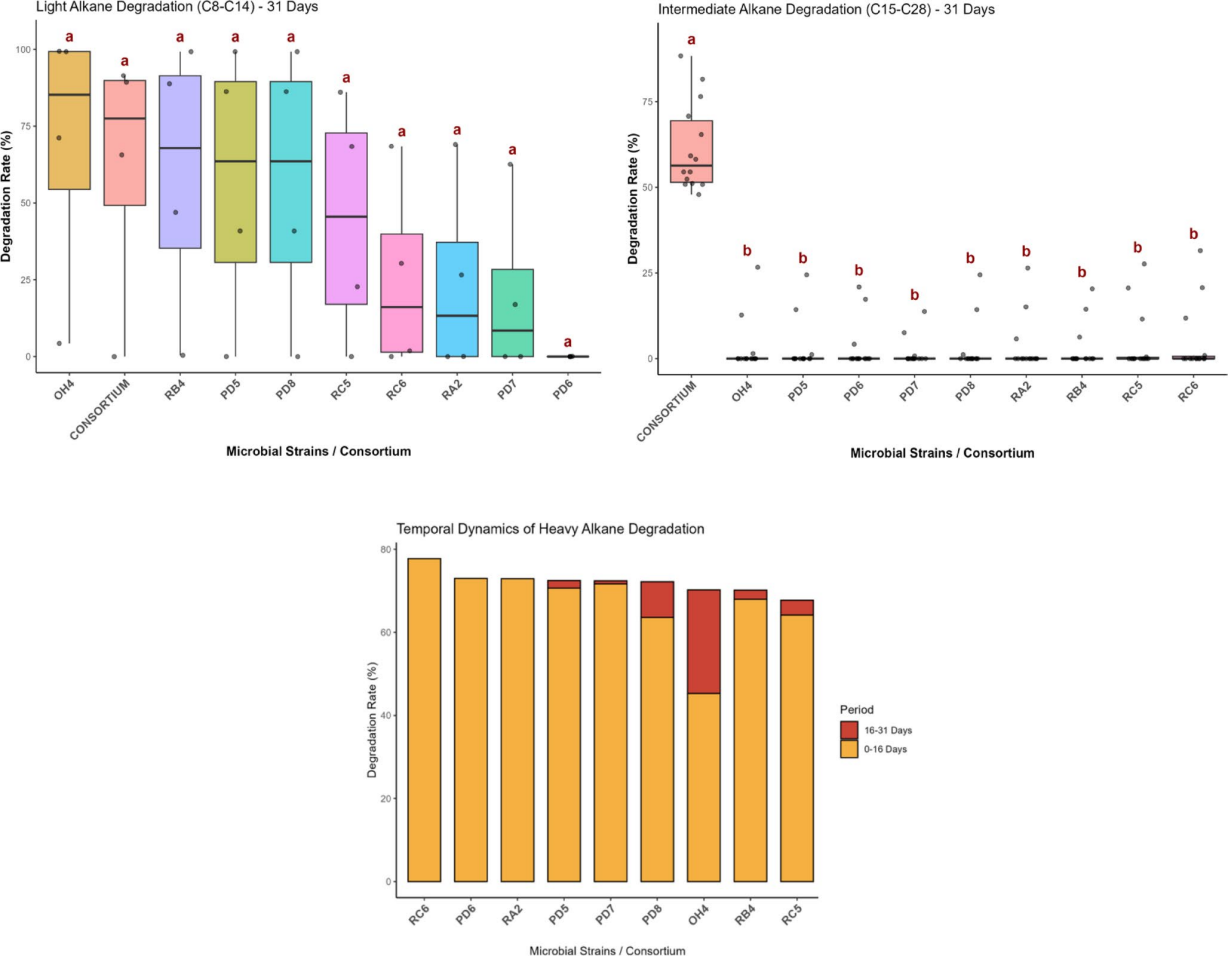
Boxplot of the degradation rates for light and intermediate alkanes, and temporal dynamics of heavy alkane degradation

In the case of the individual analyses, none of the isolated strains showed a global performance equivalent to that of the complete consortium. This is an expected result, considering that crude oil is a complex mixture of hydrocarbons with different molecular weights, which intrinsically restricts the action of isolated microorganisms [16]. Even so, highly relevant specific behaviors were observed regarding the intensity and dynamics of the degradation process (Fig. 3), revealing the metabolic specialization of the strains when interacting with particular fractions of the compounds.

Initially evaluating the light fraction, it was found that the degradation process occurred with high intensity across all treatments. All isolated strains were capable of degrading these hydrocarbons equally among themselves, reaching degradation levels statistically equivalent to those of the complete consortium. This pattern demonstrates that the metabolization of short-chain compounds is a widely shared and highly efficient baseline process among the studied microorganisms, not constituting a limiting metabolic step.

In contrast, the degradation of the intermediate fraction evidenced a completely distinct dynamic dependent on complex ecological interactions. The microbial consortium proved to be the only treatment capable of promoting statistically significant degradation (*p* < 0.05) for this range of hydrocarbons. The lack of expressive efficiency by the isolated strains in this fraction confirms that the breakdown of intermediate chains strictly requires strong metabolic synergy. Within the consortium, cooperative action allows for the cascading utilization of byproducts, preventing the accumulation of toxic metabolites that would otherwise inhibit the action of a single bacterium [22].

Regarding the heavy fraction, all isolated strains demonstrated excellent degradation rates, highlighting a strong specialization for higher molecular weight compounds. In this scope, the *Pseudomonas* and *Stenotrophomonas* genera stood out with a rapid degrader profile. This behavior is likely driven by the presence of the alkane monooxygenase enzyme, previously identified in the genomic analyses [18]. These strains exhibited a notable bimodal profile, characterized by high degradation rates for both short-chain (< C13) and long-chain (> C26) alkanes, which contrasts drastically with the sharp drop in efficiency observed in the intermediate range. Such behavior suggests that these lineages possess multiple enzymatic systems, with distinct specificities that are activated according to different carbon chain lengths [17, 29]. Conversely, all strains belonging to the *Bacillus* and *Brevibacillus* genera were positioned at the lower end of the graph regarding this rapid initial degradation profile, indicating a distinctly different metabolic timing and strategic approach to recalcitrant compounds.

**Fig. 4:**
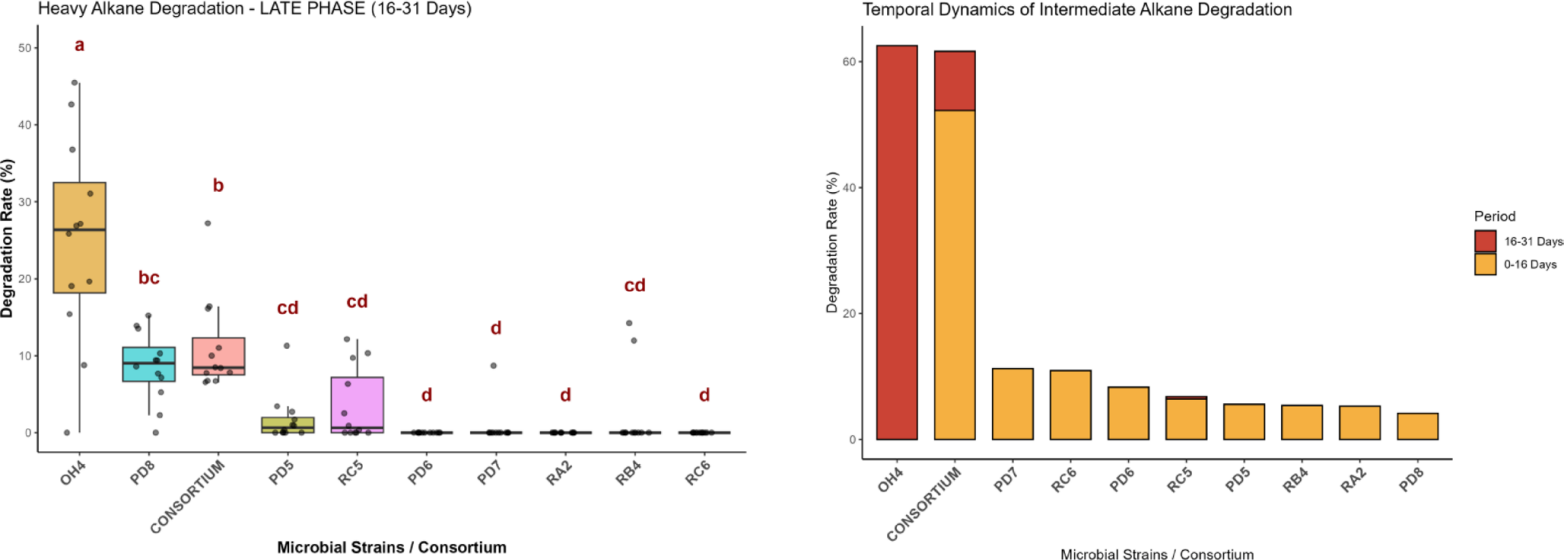
Boxplot of the heavy alkane degradation rates during the last 16 days and the temporal degradation dynamics of the intermediate fraction

On the other hand, strain OH4 (*Bacillus sp*.) served as the primary representative of a distinct metabolic group characterized by a delayed degradation profile. The temporal dynamics revealed that, for the intermediate hydrocarbons, OH4 (and, to a lesser extent, the *Brevibacillus* strains) initiated its degradative activity almost exclusively after day 16. When compared to the temporal dynamics of the complete consortium, a clear ecological succession becomes evident: the consortium’s bulk degradation occurred during the initial days, driven by rapid degraders such as *Pseudomonas* and *Stenotrophomonas*, followed by a late-stage degradation phase sustained by genera like *Bacillus*. This late activity by OH4 is particularly notable, given that the individual strains generally exhibited extremely low degradation rates for intermediate chains when cultivated in isolation.

The ecological significance of this delayed metabolism became remarkably evident in the heavy alkane fraction (> C28) during the final 16 days of the experiment. In this late phase, strain OH4 achieved a degradation rate significantly higher than the complete consortium and vastly superior to the other isolated strains (Tukey’s HSD, *p* < 0.05). This pattern of preferential and delayed consumption of complex hydrocarbons is a classic hallmark of biosurfactant-producing microorganisms. The temporal delay suggests that the bacterium initially invests energy in synthesizing and secreting biosurfactants to emulsify highly hydrophobic waxy alkanes, subsequently making them bioavailable for its degrading enzymes [7, 20, 35].

This hypothesis is strongly corroborated by previous characterizations of the OH4 strain, which confirmed its high capacity for biosurfactant production. In prior standard assays, the strain exhibited a significant emulsion index (E24) ranging from 48.6% to 57.1% against toluene. This proven physical-chemical emulsification capacity perfectly justifies OH4’s superior late-stage performance, allowing it to overcome the bioavailability limitations of heavy, recalcitrant fractions that typically restrict other non-producing microorganisms.

Beyond these temporal and functional distinctions, another critical aspect of the degradative dynamics is the high data variability observed in the boxplots for the isolated strains across both the initial and late experimental phases (Figs. 3 and 4). This wide spread of data points across specific alkane chains could initially be interpreted as extreme metabolic instability. However, considering the physical complexity of multiphasic batch systems, we hypothesize that this phenomenon, including the apparent absence of degradation or occasional anomalous values punctually higher than the initial concentration (T0), is largely driven by a methodological artifact rather than a true biological inability.

In cultures of isolated strains lacking a complete and immediate emulsification network, it is theorized that the residual oil forms heterogeneous droplets strongly adhered to biofilm aggregates on the flask walls [15]. Consequently, routine manual sampling may inadvertently capture these highly concentrated, unrepresentative ‘islands’ of sequestered oil and biomass, producing artificially inflated chromatographic peaks. This hypothesis of “floating degradation” caused by physical sequestration is supported by the comprehensive genomic repertoire for alkane degradation possessed by these strains, which contradicts such severe metabolic gaps [12, 13, 24]. To mitigate this spatial limitation and further investigate this theory, future studies should consider volumetric sampling of the entire biphasic system followed by robust ultrasound homogenization.

Interestingly, the temporal distribution of these high-variability artifacts further corroborates the biosurfactant-driven ecological dynamics proposed in this study. In strain OH4, for instance, these anomalous fluctuations were predominantly restricted to the first 16 days. This suggests that the strain initially struggled with extreme system heterogeneity; however, the subsequent accumulation of its biosurfactants likely emulsified the oil by day 31. This physical emulsification homogenized the culture medium, resulting in more precise sampling and a clear stabilization of the degradation curves in the late phase. Conversely, the complete consortium exhibited significantly less variability (narrower boxplots) from the beginning. By integrating diverse metabolic strategies and continuous surfactant production from the onset, the consortium maintained a well-homogenized multiphasic system, minimizing spatial sampling errors and ensuring consistent degradation trends.

## Conclusion

The study successfully demonstrated that the high alkane degradation efficiency of the target microbial consortium in paraffinic oil is occasioned by functional synergy and ecological niche partitioning among its members. The integration of genomic and geochemical data allowed to confirm the consortium’s catabolic potential and to assign specific roles to the most representative genera. *Pseudomonas* and *Stenotrophomonas* acted as primary and rapid degraders, while *Bacillus* specialized in the slower and later degradation of the long-chain recalcitrant compounds, probably mediated by biosurfactant production.

The geochemical results corroborated the genomic inferences and evidenced the need for a critical interpretation in multiphasic systems, since anomalous or negative degradation values reflect methodological artifacts resulting from biofilm formation and sampling heterogeneity, and not a metabolic limitation. Together, the findings reinforce that the application of microbial consortia with functional diversity constitutes a robust and adaptable strategy for the bioremediation of complex contaminants, such as petroleum alkanes.

## Data Availability

The datasets generated during and/or analysed during the current study are available from the corresponding author on reasonable request.
